# User-centered development of a personalized adaptive mirror therapy for upper-limb post-stroke rehabilitation using virtual reality and myoelectric control

**DOI:** 10.3389/fbioe.2025.1655416

**Published:** 2025-08-28

**Authors:** Paolo De Pasquale, Daniela De Bartolo, Marta Russo, Denise J. Berger, Antonella Maselli, Daniele Borzelli, Emma Colamarino, Donatella Mattia, Christian Nissler, Markus Nowak, Elena Falomo, Javier Soto Morras, Moco Raffael Schiller, Claudio Castellini, Giovanni Morone, Andrea d'Avella

**Affiliations:** ^1^ IRCCS Centro Neurolesi Bonino Pulejo, Messina, Italy; ^2^ Clinical Laboratory of Experimental Neurorehabilitation, IRCCS Fondazione Santa Lucia, Rome, Italy; ^3^ Laboratory of Neuromotor Physiology, IRCCS Fondazione Santa Lucia, Rome, Italy; ^4^ Institute of Cognitive Sciences and Technologies, Consiglio Nazionale delle Ricerche (CNR), Rome, Italy; ^5^ Department of Systems Medicine and Center of Space Biomedicine, University of Rome Tor Vergata, Rome, Italy; ^6^ Department of Biomedical Sciences, Dentistry and Morpho-Functional Imaging, University of Messina, Messina, Italy; ^7^ Department of Computer, Control and Management Engineering, Sapienza University of Rome, Rome, Italy; ^8^ Neuroelectrical Imaging and Brain Computer Interface Lab, IRCCS Fondazione Santa Lucia, Rome, Italy; ^9^ Institute of Robotics and Mechatronics, German Aerospace Center (DLR), Weßling, Germany; ^10^ NEEEU Spaces GmbH, Berlin, Germany; ^11^ Assistive Intelligent Robotics Lab, Friedrich-Alexander-Universität Erlangen-Nürnberg (FAU), Erlangen, Germany; ^12^ Department of Life, Health and Environmental Sciences, University of L’Aquila, L’Aquila, Italy; ^13^ Department of Biology, University of Rome Tor Vergata, Rome, Italy

**Keywords:** stroke rehabilitation, virtual reality, myoelectric control, user-centered design, mirror therapy

## Abstract

**Introduction:**

Cerebral stroke often results in significant motor deficits, including contralateral hemiparesis of the upper limb. Rehabilitation protocols with high-intensity and task-specific exercises can improve these deficits. Recent technological advancements in virtual reality (VR), myoelectric control, and exergames may be exploited to enhance rehabilitation effectiveness. However, novel rehabilitation approaches combining these novel methodologies have rarely been developed with the active involvement of both therapists and patients.

**Methods:**

An interdisciplinary team developed a novel system, Validation of the Virtual Therapy Arm (VVITA), for post-stroke upper-limb rehabilitation combining VR, myoelectric control, and exergames using a user-centered design (UCD) approach. The VVITA hardware includes a head-mounted VR display, motion tracking devices integrated in the VR system, and wireless armbands to record electromyographic (EMG) signals, providing an interactive virtual environment for immersive rehabilitation exercises implementing a virtual mirror therapy. Assistance and task difficulty are adjusted dynamically based on patient performance, promoting active participation and motor learning.

**Results:**

The development process involved iterative phases, involving focus groups with stroke patients, therapists, and researchers. A pilot study with four stroke survivors assessed the system’s feasibility, demonstrating its potential for personalized and adaptive rehabilitation.

**Conclusion:**

The VVITA system enhances mirror therapy by integrating VR and myoelectric control, providing a tailored approach to upper-limb post-stroke rehabilitation. The UCD approach ensured the system met patient and therapist needs, showing promise for improving motor recovery and rehabilitation outcomes.

## 1 Introduction

Cerebral stroke is one of the main causes of disability in adults and most frequently involves hemiparesis of the contralateral body. Hemiparesis can cause muscular stiffness and other impairments to the fine and global motor coordination of the upper limbs, including restricted range of motion of the arm’s joints which hinders reaching and grasping movements, essential to perform daily living activities (Hatem et al., 2016).

These motor deficits occur for 80% of patients in the acute phase and for 40% of patients in the chronic phase and can be improved through rehabilitation protocols with high-intensity and/or task-specific exercises (Cramer et al., 1997; Kwakkel et al., 2004; Veerbeek et al., 2014). Technological advances in recent years have provided new methodologies to support and foster the rehabilitation process by increasing its repeatability and intensity (Foley et al., 2012; Cikajlo et al., 2020; Ceradini et al., 2024; De Luca et al., 2024), including but not limited to virtual reality (VR), myoelectric control, and exergames.

Rehabilitation systems integrated into VR systems can provide training scenarios that are more complex and engaging than those employed in conventional therapy (CT) (Maggio et al., 2023). In addition, activities of daily living (ADL) can be simulated in an ecological and controlled manner thanks to the immersivity of head-mounted displays, large projection screens, and VR caves. Thus, VR scenarios effectively increase patient’s active participation and motivation leading to a better adherence to the rehabilitation protocol (Domínguez-Téllez et al., 2020). Immersive VR systems currently available in consumer electronics and gaming markets can also record movement kinematics and allow quantitative monitoring of the motor performance of the rehabilitation exercise (De Pasquale et al., 2024). Affordable prices and ease of use make these systems suitable for home rehabilitation after hospitalization using a telemedicine approach (Piron et al., 2009).

Myoelectric control is a novel and promising approach for rehabilitation. Myoelectric interfaces have been mostly used for the control of actuators such as exoskeletons or prostheses (DiCicco et al., 2004; Liarokapis et al., 2013; Nissler et al., 2019). Thanks to myoelectric interfaces, which decode the patient’s intention through the residual myoelectric activity in the paretic limb, patients may generate voluntary movements through their spared cortico-spinal pathway and receive feedback (e.g., a realistic visual feedback from an embodied limb in VR) thus establishing a closed-loop system that promotes re-learning and encourages active participation, increasing motor coordination, muscle strength and reducing spasticity (Song et al., 2013; Sarasola-Sanz et al., 2018). The use of myoelectric interfaces for rehabilitation also aims at promoting neuroplasticity to reshape neuromuscular activity and to enhance motor learning, and the restoration of motor function. For instance, stroke patients can learn to control, with the more affected upper limb, a multi-degree-of-freedom exoskeleton using a decoder trained with EMG from the healthy limb (Sarasola-Sanz et al., 2022). Moreover, myoelectric control training can reduce abnormal co-activation (i.e., undesired coupling) by training only the desired muscles while leaving other muscles unaffected (Seo et al., 2022).

Virtual Reality and active video games that combine physical activity with interactive serious games, or exergames, have been used for rehabilitation (Maggio et al., 2020; Morone et al., 2024). In addition, a serious game was recently approved by the FDA (Food and Drug Administration) as the first digital drug in children affected by ADHD (Attention Disorder Hyperactivity Disorder) (Laver et al., 2017; Commissioner, 2020; Mubin et al., 2022). Exergames make rehabilitation exercises more enjoyable and engaging. However, exergames commercially released often are not tailored to the specific needs and requirements of patients and cannot easily incorporate feedback and suggestions from therapists. In contrast, recent advancements in assessment methodologies can be exploited to devise personalized rehabilitation approaches, aimed at restoring specific components of the motor deficits. For instance, such approaches may target the motor functionality of the proximal upper limb (shoulder and elbow joints), the distal extremities (wrist and fingers), both simultaneously, or they may focus on restoring balance or reducing treatment time (Henrique, Colussi, and De Marchi, 2019). Customized approaches also allow to take into account patients’ needs and to modify the level of difficulty of the exercise considering the patients’ functional assessment, ability and capability, and the achieved level of motor recovery, which are not usually quantified in commercial game-based applications (Tsekleves et al., 2016; Han et al., 2017; Triandafilou et al., 2018; Zirbel et al., 2018). Customization also allows to directly involve therapists in the development of rehabilitation systems regarding the definition of system requirements including difficulty to use, time to set the system and the required knowledge (Rand et al., 2018).

Indeed, while VR, myoelectric control, and exergames represent promising methodologies that can be exploited to introduce novel rehabilitation therapies, the active involvement of both therapists and patients during the whole development process is critical to improve their usability and effectiveness. In fact, a user-centered design (UCD) approach is increasingly used to develop, improve, and evaluate new rehabilitation systems or procedures. This approach may also facilitate system usage and integration in clinical and domestic settings thus overcoming the limitations of current approaches (Ríos-Hernández et al., 2021; Semprini et al., 2022).

The goal of the present work is to present the development of a system combining VR and myoelectric control to implement a new mirror therapy (MT) approach for post-stroke upper-limb neuromotor rehabilitation. MT is a rehabilitation technique that has significant potential to be enhanced by exploiting new methodologies such as VR, myoelectric control, and personalized exergames through a UCD development process. During conventional MT patients watch their unaffected limb reflection in a mirror placed on a sagittal plane between patients’ limbs. The reflection of the unaffected limb in the mirror creates the illusion that the affected limb moves effectively and painlessly and provides encouragement. MT was first developed to alleviate phantom limb discomfort in persons with amputation (Ramachandran and Rogers-Ramachandran, 1996), and then applied to stroke patients with weaker limbs to improve muscle control (Altschuler et al., 1999). According to a systematic review, MT is recommended as a valuable approach to be integrated in the rehabilitation intervention of stroke patients (Hatem et al., 2016). However, conventional MT has a few limitations that restrict its use in clinical settings including being monotonous, only providing a low-dose therapy, and requiring specialized equipment and a professional on-site (Horne et al., 2015). Moreover, movements are limited by the physical dimension of the “mirror box”. By allowing for more clinically viable use of MT approach, VR-based therapy may overcome these restrictions and motivate patients to perform rehabilitation protocol including meaningful ADL tasks. VR is indeed seen as a potential method for delivering larger therapeutic dosages and enhancing post-stroke arm/hand rehabilitation (Chen et al., 2015).

Arm-hand movement training in VR is a successful technique for increasing the functional motor recovery of stroke patients, thanks to VR granting the possibility to enhance multisensory feedback in a highly controllable and versatile fashion (Laver et al., 2017; Massetti et al., 2018). The mirror visual illusion that appears in VR systems facilitates the multisensory integration by promoting the interaction between bilateral proprioceptive signals and visual input (Giroux et al., 2018). Recent studies show that, combining VR with MT leads to better rehabilitation results (Perez-Marcos et al., 2018), especially when using a contralateral lesion action observation network (Saleh et al., 2017). Although it has been already proven that VR technology positively affects motor functionality delivering highly immersive environments for motor learning, the use of VR with MT protocols have so far shown limited evidence of effectiveness due to the small number of recruited patients, an inadequate research design and/or low-intensity training (Hsu et al., 2022).

Therefore, we developed a novel system to overcome the limitations of a conventional MT by exploiting the potentiality of VR to enhance the effectiveness of rehabilitation. Specifically, the system allows patients suffering from stroke upper-limb hemiparesis to control the related virtual paretic arm thanks to an innovative control algorithm based on input from both limbs whose relative contribution is modulated adaptively by monitoring the progress of patients. Here, we present the UCD of the system, from the initial concept that emerged within our interdisciplinary research team, through a series of development and validation steps, up to the definition of a specific system configuration and training protocols to be evaluated systematically in future studies.

Using the UCD approach, we developed the Validation of the Virtual Therapy Arm (VVITA) system for upper-limb post-stroke rehabilitation starting from the existing Virtual Therapy Arm (VITA) system, designed for treating phantom-limb pain in people with limb-loss and performing prosthetic training (Nissler et al., 2019). The development process included focus group sessions with clinical and motor control experts to ensure the translation of engineering outcomes into clinical practice. Hence, the definition of the new control modality of the system to be used with stroke patients through intermediate assessments and, where needed, the refinement of the design. In summary, although previous studies have explored VR-based mirror therapy and myoelectric interfaces separately or in combination, our system presents distinctive innovations. First, we propose an adaptive bimanual control algorithm that dynamically integrates EMG signals from both upper limbs, modulating their relative contribution according to individual patient progress. This allows a personalized progression of training intensity and supports active engagement of the paretic limb beyond traditional unimanual or fixed-threshold approaches. Furthermore, the system was developed following a rigorous user-centered design process, involving clinicians and therapists throughout all stages of development to ensure clinical relevance, usability, and effective integration into rehabilitation practice. These features represent a novel contribution to the field by addressing key limitations of conventional mirror therapy and previous VR-EMG systems. Finally, we present the results obtained from a pilot study involving four stroke survivors, providing an initial assessment of the feasibility of our novel personalized adaptive mirror therapy approach for upper-limb post-stroke rehabilitation based on virtual reality and myoelectric control.

## 2 Methods

The VVITA system has been developed as a stroke rehabilitation application of the VR platform originally developed for the treatment of phantom limb pain in upper-limb amputees within the VITA project led by a research group at the Institute of Robotics and Mechatronics of the German Aerospace Center (DLR) in Munich, Germany. The VITA system originally developed for amputees has been modified to be used with post-stroke patients to rehabilitate the neuromotor functionality of the more affected upper limb. This development involved an interdisciplinary and international team composed of several research groups. The DLR group modified the system according to suggestions provided by two groups at Fondazione Santa Lucia (FSL) in Rome, Italy, including motor control and rehabilitation experts. Following an iterative UCD approach, the team specified the requirements for new training protocols for upper-limb post-stroke neurorehabilitation, implemented those protocols in the new software named VVITA, developed methods for their assessment, and performed several evaluations of the new system by testing it in a pilot study with stroke patients.

### 2.1 The VITA system

The VITA system (Nissler et al., 2019) is a low-cost VR solution developed to treat phantom-limb pain of people with limb-loss or to perform prosthetic training. It uses an HTC *Vive Pro* VR platform (HTC Europe Co. Ltd., Slough, Berkshire, United Kingdom), including a head-mounted display and two trackers, and two *Myo* armbands (Thalmic Labs, Ontario, Canada) with EMG sensors. One tracker and one armband are placed on each amputee’s arm. One of the Vive Trackers is placed on the unimpaired hand dorsum and provides the position and orientation of the hand. The second tracker is placed on the remaining portion of the amputated limb to provide its position and orientation. The Myo armband consists of eight bipolar surface EMG sensors measuring the activity of several forearm muscles. The sensors are mechanically connected and arranged in an armband placed on the amputee’s forearms to record most of the muscle controlling opening and closing of the fingers. A representation of the hands is provided in the virtual environment as visual feedback. Virtual hand movements are directly predicted from the kinematic recorded with the trackers while virtual finger movements are predicted from the forearm muscle signals using a model trained through a machine learning procedure. The machine learning method, an iterative variant of Random Fourier Features Ridge Regression (iRR-RFF) (Patel et al., 2017), trains a non-linear decoder mapping features from the EMG signals onto finger gestures and used to control the feedback given by the virtual hands. The acquired signals (kinematic and EMG) are wirelessly transmitted via Bluetooth technology. Data (kinematic and EMG signals) acquisition, model training, and prediction processes are performed by a laptop (Alienware m15, Dell) with a dedicated GPU (GeForce RTX 2060, Nvidia). The virtual scene reproduces a house surrounded by nature (trees, lake, mountains, etc.), in which various activities both inside the house (cooking, playing the drums, stoking up a fireplace, etc.) and external (picking fruit, etc.) can be carried out for rehabilitation purposes. Participants can navigate within the virtual environment by moving to the desired area according to the exercise they choose to perform.

### 2.2 UCD approach

A UCD approach was implemented in the VVITA project to develop a novel application of the VITA system optimized for stroke rehabilitation, involving a multidisciplinary team of biomedical engineers, motor control neuroscientists, neurologists, therapists, and patients. A focus group, including seven stroke patients, one physiotherapist, one psychologist, one specialist in physical medicine and rehabilitation, one neurologist, five motor control scientists, and five engineers supported the protocol development. This team brought expertise in neurological disorders, VR-based therapies, and neuromotor rehabilitation to ensure the system met both clinical and patient needs. The technical development also involved NEEEU (NEEEU Spaces GmbH, Berlin, Germany), a company focused on applying Human Centered Design processes to new technologies, which collaborated as subcontractor on the design, qualitative research and development of the patient and therapist journeys, the virtual environment and the digital therapy platform for therapists. For these developments, we specifically used a service design–centered approach, leveraging tools such as journey maps and service blueprints to map the user’s experience over time. By combining these methodologies, we ensured that the evolving rehabilitation service was continuously refined in collaboration with end users.

**FIGURE 1:**
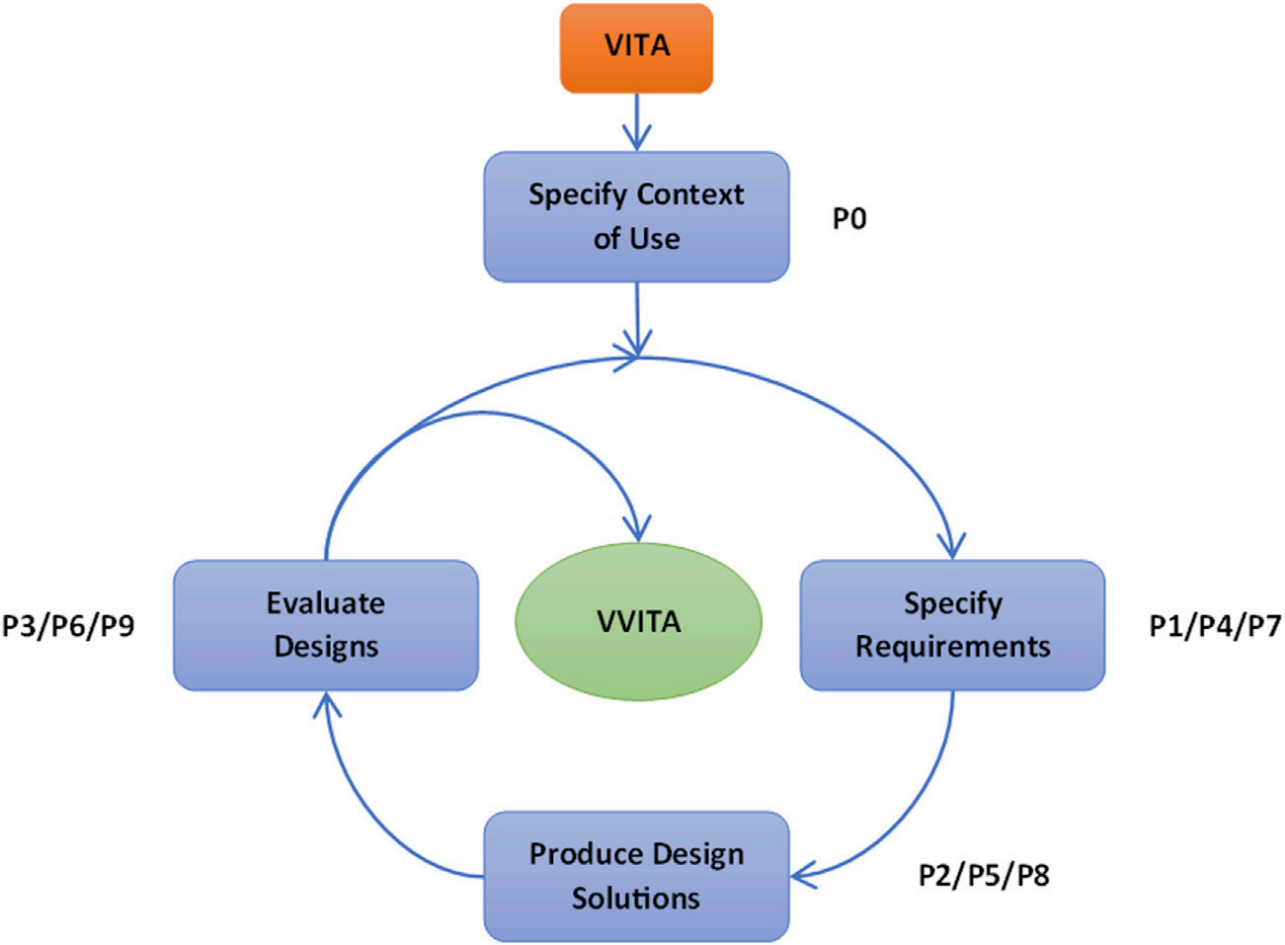
Schematic of the UCD process. The figure illustrates the iterative approach of the UCD methodology, detailing the various phases (P) that transformed the initial VITA into the final VVITA system. The phases specify requirements, produce design solutions and evaluate designs were repeated three times to refine the system and address issues identified during testing.

The development activities followed three iterative cycles, each composed of distinct phases (P). The first cycle included four phases: (0) evaluation of the system’s context of use, included only in first the cycle, (1) specification of the rehabilitation protocol based on experts’ requirements, (2) software development to implement the desired protocol, and (3) system evaluation with healthy participants and stroke survivors. The initial phase (P0), focused on assessing the system’s intended context of use, defining scientific questions, and identifying desired outcomes. Inputs from this phase guided the specification of system requirements and training protocols in the subsequent phase (P1). The first version of the software was developed in the third phase (P2) and it was critically tested and evaluated during the fourth phase (P3). In the two additional cycles, the first, second and third phases were repeated to refine the protocol, address updated system requirements, incorporate feedback from therapists and patients, and resolve issues identified during development (Figure 1).

The first cycle (P0, P1, P2, P3), lasted 30 months and consisted of 40 interactions, the second cycle (P4, P5, P6) lasted 2 months and consisted of 13 interactions, while the last cycle (P7, P8, P9) lasted 4 months and consisted of 12 interactions (See Table 1).

**TABLE 1:** Summary of UCD phases and cycles**.** The phases included in each cycle of the UCD approach: the type of phase, number of iterations conducted within each phase, and a brief description of their objectives and outcomes.

Cycle	Stage	Type	Number of interactions	Description
1	P0	Meetings	7	Specify Context of Use
	P1	Meetings	23	Specify Requirements
	P2	Meetings	4	Produce design solution
	P3	Testing healthy subjects	6	Evaluate designs
2	P4	Meetings	7	Specify Requirements
	P5	Meetings	3	Produce design solution
	P6	Pre-pilot stroke patients	3	Evaluate designs
3	P7	Meetings	6	Specify Requirements
	P8	Meetings	2	Produce design solution
	P9	Pilot stroke patients	4	Evaluate designs

The final software release addressed all the issues that had emerged during the development, and it was tested in a pilot study (P9) involving four stroke patients (two chronic and two sub-acute). This study evaluated the system’s feasibility, functionality, and usability through kinematic analysis and user feedback, demonstrating the system’s potential for enhancing post-stroke rehabilitation.

### 2.3 Incremental development and testing

The VVITA system was developed following a structured UCD approach. Each phase was designed to iteratively refine the system to meet the specific needs of post-stroke upper-limb rehabilitation. In this section we describe the main activities performed in each phase of development. The outcome of these activities, including the novel VVITA system and the results of the pilot study, are presented in the Results.

#### 2.3.1 P0 – Specification of the context of use of the VVITA system

The first phase defined the context of use, targeting subacute and chronic stroke patients with diverse motor impairments. Inclusion criteria addressed motor deficits, age, and cognitive ability to ensure compatibility with the system. Patients undergoing treatments like botulinum toxin were excluded if such treatments occurred 2 weeks before or during rehabilitation. A comprehensive review of the literature (Eng et al., 2007; Burke et al., 2009; Cheung et al., 2009; Dohle et al., 2009; Bohil et al., 2011; Lee et al., 2011; Thieme et al., 2012; Zhang and Zhou, 2012; Li et al., 2014; Pollock et al., 2014; Ballester et al., 2015; Choi et al., 2016; Hatem et al., 2016; Hoermann et al., 2017; Kim, 2017; Laver et al., 2017; Sadarangani et al., 2017; Darbois et al., 2018; Morone et al., 2019) identified limitations in existing systems, such as inadequate adaptability and underutilization of immersive VR and myoelectric control. These findings guided the conceptual design of the VVITA system, which integrated VR mirror therapy, myoelectric control, and dynamic task adjustments to enhance patients’ engagement. Focus group discussions refined these concepts, emphasizing patient motivation, adaptive protocols, and therapist control. These inputs formed the foundation for subsequent phases.

#### 2.3.2 P1 – Definition of the system requirements and specifications

Building on the outcomes of Phase P0, detailed requirements were established for the hardware, software, and training protocols. The hardware design included motion sensors for hands’ tracking and EMG sensor bracelets for myoelectric control. The software specifications encompassed immersive VR environments, calibration procedures, and assistive control algorithms for proximal (shoulder, elbow) and distal (hand) joints. Training protocols were designed to include bimanual reaching and grasping tasks tailored to individual motor capabilities, with dynamically adjustable difficulty. The aesthetics of the platform were carefully chosen to avoid an uncanny-valley effect, virtual objects and hand representations are stylized rather than hyperrealistic, while still providing an immersive experience. A graphical user interface (GUI) for therapists was found to be essential for enabling real-time customization of parameters and monitoring of patient progress. Notably, therapists can visualize patients’ actions in VR through a dedicated monitor, allowing them to observe kinematics movements and EMG-driven gestures live as part of the feedback mechanism. An initial GUI design underwent a 30-min click dummy evaluation followed by therapist feedback, ensuring alignment with patients’ needs. This phase produced the initial system architecture, outlining the integration of hardware components, software modules, and control algorithms, providing the basis for Phase P2.

#### 2.3.3 P2 – First software release

The first software version was developed based on the specifications defined in Phase P1. Key features included calibration tools, movement control strategies, task design, and real-time feedback. The calibration tools ensured alignment between the virtual and physical setups, enabling precise interaction with the virtual environment and tailoring experimental parameters to the patient’s motor capabilities. The movement control strategies allowed the virtual more affected limb (VMAL) to be driven by the patient’s residual motor capabilities, with dynamic adjustable parameters to mirror the movements of the real less affected limb (RLAL) to amplify the movements of the real more affected limb (RMAL). The task design incorporated bimanual reaching and grasping exercises, carefully designed to offer engaging and challenging experiences while promoting motor recovery. Real-time feedback mechanisms delivered visual and auditory cues to guide task execution and enhance patient engagement. This version served as a proof of concept, demonstrating the feasibility of integrating virtual reality, motion capture, and myoelectric control into a cohesive rehabilitation platform.

#### 2.3.4 P3 – Initial evaluation of the system by healthy participants and therapists

Healthy participants tested the system under therapist supervision to identify usability and functionality issues. The evaluation aimed to highlight limitations and challenges in calibration and target placement procedures, as well as to identify potential compensatory motor strategies exhibited by patients during tasks. Furthermore, the test supported therapists to identify the range of task parameters and type of feedback mechanisms compatible with the intended applications.

#### 2.3.5 P4 – Refinement of the system requirements and specifications

Based on the findings from Phase P3, several refinements were implemented to optimize the system for usability and adaptability across diverse rehabilitation needs. Calibration procedures were enhanced to improve alignment accuracy, while target placement algorithms were revised to accommodate varying ranges of motion. Task parameters were adjusted to balance challenge and fatigue. For example, grasping holding times were shortened. An adjustable table was integrated to enhance comfort and accessibility for patients of different sizes and mobility levels. Additionally, control algorithms were refined to ensure smoother transitions between mirrored and independent movements. These updates optimized the system for usability and adaptability across diverse rehabilitation needs.

#### 2.3.6 P5 – Second software release

The second software release resolved the issues identified in Phase P3 and incorporated updates based on the refined system requirements from Phase P4. Key improvements included a configuration file that enabled therapists to customize experimental parameters prior to sessions, such as spatial tolerance for successful target reach, maximum trial duration (timeout), and trial success time (holding time required to successfully reach and perform a gesture). Visual and auditory feedback mechanisms were enhanced for better clarity and responsiveness. Additionally, adjustments to target orientation and placement were made to address positioning issues, such as interactions with the table or targets placed in physiologically challenging positions, ensuring better accessibility without compromising therapeutic effectiveness. These updates marked a significant advancement, addressing limitations from earlier phases and preparing the system for evaluation with stroke patients.

#### 2.3.7 P6 – Evaluation of the system by therapists and stroke patients

The revised software was tested with three chronic stroke patients during a pre-pilot assessment to evaluate usability and gather additional feedback. Patients with varying levels of impairment participated in multiple sessions (Table 2), during which therapists dynamically adjusted parameters to tailor tasks to each patient’s individual capabilities. Observations and feedback from both therapists and patients were instrumental in informing further refinements in the subsequent development phase.

**TABLE 2:** Participants included in the pre-pilot usability assessment. Demographic and clinical data of the participants to the pre-pilot assessment.

Patient	Age	Gender	Stroke type	More affected limb	Time since stroke (years)	Session N°	FMA score
1	33	Male	Hemorrhagic	Right	0.4	4	56
2	67	Female	Ischemic	Left	18.92	3	18
3	41	Female	Ischemic	Left	4.47	1	36

#### 2.3.8 P7 – Second refinement of the system requirements and specifications

Based on the feedback gathered during Phase P6, several refinements were identified as required to enhance the system’s usability and clinical effectiveness. These included updating the virtual hand design, optimizing the virtual environment, refining target placement algorithms, and addressing issues related to compensatory movement strategies. Updating the virtual hand design involved enhancing its color and appearance to improve patient embodiment and reduce confusion during tasks. Optimizing the virtual environment focused on removing non-interactable objects from the virtual environment to minimize distractions and improve task focus. Refining target placement algorithms aimed to improve accessibility, particularly for lateral targets, while maintaining their therapeutic value. To address the issues identified with the presence of compensatory movements it was decided to optimize the control algorithms and to provide therapists with tools to better manage these behaviors. Additionally, the need to develop a comprehensive operation manual to assist therapists in system calibration, parameter adjustments, and managing compensatory movement strategies effectively was identified. These refinements were deemed critical to delivering a more effective, intuitive, and engaging rehabilitation platform.

#### 2.3.9 P8 – Final software release

The final version of the system successfully addressed all the remaining usability issues, incorporating feedback and improvements to enhance its effectiveness. Key refinements included embodiment improvements, with optimized virtual hand designs for better interaction and engagement, and therapist interface enhancements, featuring improved GUI functionality to allow seamless real-time adjustments of parameters. Calibration refinements ensured consistent accuracy through improved alignment procedures.

While trunk compensation remained a challenge, therapists were equipped with tools and guidelines to manage these behaviors effectively. A comprehensive user manual was also provided to therapists prior to the pilot experiment. The manual detailed the hardware and software components of the system, described operating procedures, outlined the initial calibration process, and explained policies for adjusting assistance parameters during training. This version was deemed ready for clinical evaluation, incorporating critical updates and support materials to facilitate effective use and study of the system in real-world scenarios.

#### 2.3.10 P9 – Evaluation of the system with a pilot study

Phase P9 involved a pilot study designed to evaluate the feasibility, usability, and clinical impact of the final VVITA system. Four stroke patients were recruited for this phase, including two in the chronic phase (at least 1 year post-stroke) and two in the subacute phase (less than 1 year post-stroke) (See Table 3).

**TABLE 3:** Participants included in the pilot study. Demographic and clinical data of the participants to the pilot assessment.

Patient	Age	Gender	Stroke type	More affected limb	Dominant hand	Time since stroke (months)	Session N°	FMA score
E1	39	Female	Ischemic	Left	Right	48.7	13	38
E2	65	Male	Ischemic	Right	Right	284.3	12	24
E3	72	Male	Ischemic	Left	Right	0.8	4	16
E4	77	Female	Ischemic	Right	Right	0.5	2	18

To avoid confounding factors, participants had not received additional treatments, such as botulinum toxin, in the 2 weeks prior to or during the study. The study was conducted in accordance with the Declaration of Helsinki and approved by the ethical review board of FSL (Prot. CE/PROG.790). Written informed consent was obtained from all participants before the experimental sessions began.

The pilot study lasted 1 month, with each participant completing rehabilitation sessions three times per week. Tasks involved bimanual reaching and grasping, calibrated to each patient’s individual movement range. Real-time feedback was provided during the tasks to guide performance, while therapists dynamically adjusted assistance parameters and task difficulty to ensure the optimal balance between challenge and feasibility. The primary focus was to evaluate the system’s ability to support motor recovery and maintain patient engagement throughout the rehabilitation process. Validated questionnaires were used to assess the system’s usability, feasibility and patient experience.

For the evaluation of the usability, the feasibility of the developed rehabilitation system and patient experience, the following questionnaires were administered to the pilot experiment participants: the User Satisfaction Evaluation Questionnaire (USEQ, (Gil-Gómez et al., 2017)), for evaluation of the evaluation of the VR system usability with range from 6 to 30; the Visual Analogical Scale (VAS) with range from 0 to 10 with respect to subjective motivation and satisfaction related to exercise; the Pittsburgh Participation to rehabilitation Scale (PPRS, (Iosa et al., 2021)) compiled by the researcher/therapist to report the patient’s participation levels in the exercise on a Likert scale ranging from 1 to 6 and the National Aeronautics and Space Administration Task Load Index (NASA-TLX, (Hart and Staveland, 1988)) for the multidimensional subjective assessment that rates perceived workload to assess a task, system, or team’s effectiveness or other aspects of performance.

The Fugl-Meyer Assessment (FMA) for the upper limb was used to evaluate motor recovery and functional ability. This evidence-based scale (Deakin et al., 2003) assesses motor recovery across multiple stages, with individual items scored on an ordinal scale from 0 (unable to complete the task) to 2 (successfully completed). The FMA provided a standardized measure of the system’s effectiveness in promoting motor improvements.

Instrumental data were recorded through the VVITA system’s integrated sensors. The recorded kinematic from the hands (3D position and rotation the back of the hand) was tracked at 30 Hz, filtered using a fourth-order Butterworth low-pass filter with a 3 Hz cutoff frequency, and analyzed so to extract assessment variables such as performance scores, assistance parameters, maximum speed, and range of motion (ROM).

Both clinical and instrumental evaluations were conducted at the beginning (T0) and end (T1) of the rehabilitation protocol to assess the system’s impact. Feedback from patients and therapists was also collected after each session to identify any remaining usability challenges and gauge overall satisfaction with the rehabilitation experience.

### 2.4 Statistical analysis

We evaluated the variation in overall assistance level, Fugl-Meyer index, and maximum speed across sessions (*Se*) using a linear mixed model (LMM). This model accounted for interindividual variability by including participants as a random effect. The experimental factor (*Se*) was treated as a fixed effect with categorical (dummy) variables. Data were fitted with the model in Equation 1:

$$Y=u_{0}+\alpha_{0}Se+\epsilon$$ (1)

where $u_{0}$ is the individual intercept and accounts for inter-individual differences, $\alpha_{0}$ is the fixed-effect slope, thus the modulation of the response variable by the factor Se . As data represent a continuous variable, they were fitted with a LMM (Matlab, function *fitlme*). Estimation of model parameters were based on the maximum likelihood using Laplace approximation.

## 3 Results

A UCD approach was employed to develop a novel system for upper-limb post-stroke motor rehabilitation by modifying a system initially created to treat phantom limb pain in amputees. The training activities, assistive algorithms and user interface were developed and refined through 65 interactions among all the members of the development team, achieving a synthesis between technological innovation and rehabilitation principles.

Following an initial analysis of the patients’ needs and rehabilitation goals, a bimanual reaching task was selected as the primary training activity. Virtual mirroring (Saleh et al., 2017; Giroux et al., 2018; Mekbib et al., 2021; Hsu et al., 2022; da Silva Jaques et al., 2023), using both kinematic signals for hand position and EMG signals for hand gesture, was defined as the key assistive approach to enhance functional recovery. Initially, 3 different control algorithms were considered to provide virtual mirroring of the more affected hand. These algorithms were implemented for pilot testing, each affecting the control modality and the guidance of the VMAL differently, based on the RLAL and the RMAL.

The amount of mirrored assistance provided to the VMAL, and the task difficulty were controlled by two parameters: an agency parameter ( α ) and a capability parameter ( β ) for both the distal and proximal components of the more affected limb. The outcomes of each development phase, as well as the results of the final evaluation of the system with a pilot study are reported in the following two sections. For the pilot study, metrics related to system usability, user experience and task performance are reported.

### 3.1 Outcomes of incremental development

#### 3.1.1 VVITA system

**FIGURE 2:**
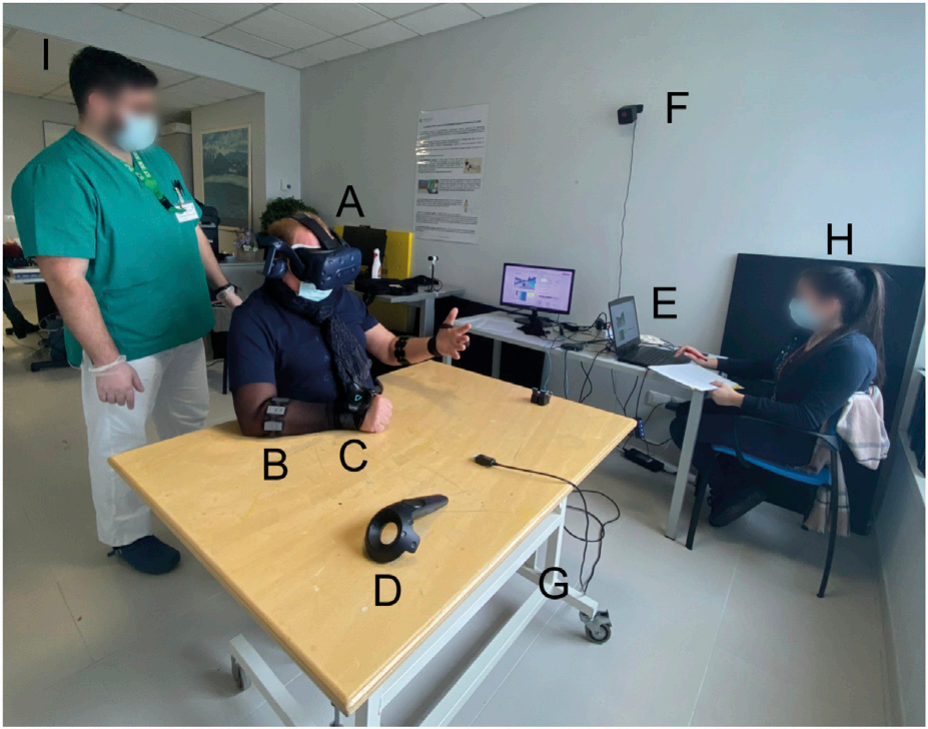
VVITA Setup. **(A)** Virtual reality headset *Vive* by HTC. **(B)** EMG bracelet *Myo* by Thalmic Labs. **(C)** HTC tracker. **(D)** HTC controller. **(E)** Laptop. **(F)** One of the two HTC Vive base stations. **(G)** Adjustable table. **(H)** Therapist. **(I)** Physiotherapist.

The VVITA system is based on the VITA system with which it shares the hardware architecture. Differently from the VITA system, in VVITA both trackers are placed on the dorsum of the patient’s hands (Figure 2). Several software solutions were developed and adopted to provide a virtual mirror therapy assisted by the hand kinematic and gesture prediction of the less affected arm depending on the level of impairment and the functional restoration achieved during the treatment. The system allows a patient to practice rehabilitation exercises that simulate the performance of ADL in an immersive VR environment, providing real-time visual feedback of a bimanual reaching and grasping task in which the movement of a virtual limb reproduces and improves the movement of the paretic limb. The movement of the VMAL is displayed based on the movement and the EMG activity of both the RMAL and the RLAL recorded with the system integrated motion capture sensors and the armbands as in the original VITA system.

#### 3.1.2 Assistive and control algorithms

The VVITA system introduces innovative assistive and control algorithms that enable virtual mirror therapy, a functionality not available in the original VITA system. The system supports bimanual rehabilitation tasks by leveraging VR capabilities to provide mirrored assistance for both limb movements (proximal control) and hand gestures (distal control). Proximal control utilizes kinematic data recorded by motion trackers, while distal control uses EMG data acquired from the Myo armband.

**FIGURE 3:**
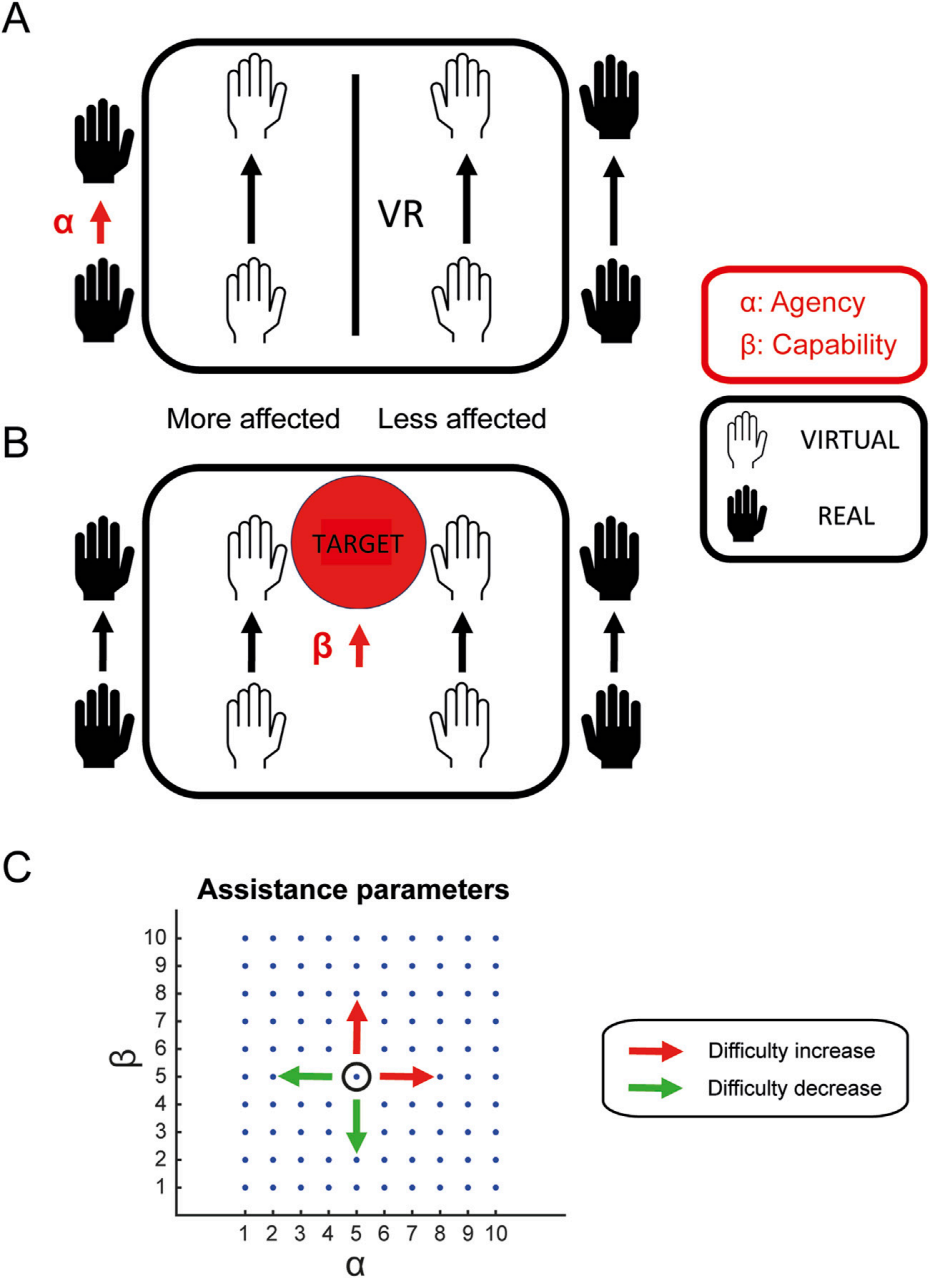
Proximal assistance. **(A)** The agency parameter α modulates the contribution of the RLAL and RMAL to the motion of the VMAL. **(B)** The capability parameter β tailors the task based on the patient’s residual motor abilities adjusting target placement distance. **(C)** A graphical representation of the relationship between α and β : higher values of α and β denote more difficult tasks due to reduced assistance and/or larger workspace.

To achieve precise control of the VMAL, the system employs an agency parameter ( α ) (Figure 3A), which determines the contribution of the RLAL and RMAL movements to the VMAL movement. This parameter can be adjusted independently for proximal ( $\alpha_{p}$ ) and distal ( $\alpha_{d}$ ) control. For proximal control, the VMAL position in 3D space is calculated as a weighted combination of RMAL and mirrored RLAL movements, defined as:

$$x_{VMAL}=\alpha_{p}x_{RMAL}+\left(1-\alpha_{p}\right)F\left[x_{RLAL}\right]$$ (2)

In Equation 2, x represents the pose (i.e., position and orientation) of the hand in Cartesian coordinates, and $F\left[x\right]$ is a mirroring function that can be defined according to different methods (see below).

For distal control, the VMAL hand gesture is determined by the EMG activity of the RMAL and RLAL:

$$q_{VMAL}=\alpha_{d}q_{RMAL}+\left(1-\alpha_{d}\right)q_{RLAL}$$ (3)

In Equation 3, q denotes a parameter controlling the degree of hand opening and closing, representing the gesture of the hand (i.e., the set of finger joint angles describing the configuration of the fingers relative to the palm), as estimated from EMG signals.

As $\alpha_{p}\approx 1$ or $\alpha_{d}\approx 1$ , the VMAL replicates the RMAL’s movement or gesture, providing complete agency and no assistance. Conversely, as $\alpha_{p}\approx 0$ or $\alpha_{d}\approx 0$ , the VMAL reflects the RLAL’s motion or gesture, resembling conventional mirror therapy. This flexibility allows the therapist to customize the assistance levels according to the patient’s motor capabilities and the success rate of reaching tasks, choosing within ten predefined assistance levels ranging from zero assistance to full assistance.

The mirroring function F in Equation 2 transforms spatial positions to compute a reference position that helps guide the movement of the VMAL based on the motion of the RLAL. Specifically, the VMAL hand is displayed at a weighted average between the RMAL hand position and the mirrored reference position. Three different methods were implemented to determine such reference position by transforming the position of the RLAL: stiff coupling, rubber band, and reference trajectories. In the stiff coupling method, the reference position is computed rigidly as the RLAL hand position mirrored across a vertical plane. The rubber band method mirrors the RLAL’s movement across the mid-sagittal plane, mimicking classical mirror therapy. The reference trajectories method maps the reference position along predefined paths derived from the pre-recorded RLAL trajectories, providing a structured reference trajectory for the more affected limb. These algorithms ensure adaptable, task-specific assistance, facilitating rehabilitation tailored to each patient’s motor recovery.

In addition to agency parameters, the system incorporates capability parameters ( β ) to adjust task difficulty dynamically (Figure 3B). The proximal capability parameter ( $\beta_{p}$ ) regulates target placement, with levels ranging from 0 (targets placed within the RMAL’s reachable range) to 1 (targets placed at the RLAL’s maximum range). Similarly, the distal capability parameter ( $\beta_{d}$ ) adjusts the tolerance for successful hand gestures, requiring minimal muscle activation if $\beta_{d}\approx 0$ and close-to-maximal effort if $\beta_{d}\approx 1$ .

The relationship between the assistance parameters and task difficulty is defined such that higher parameter values corresponded to reduced assistance levels, thereby increasing task difficulty. Specifically, as α or β increase, patients are required to rely more on their own motor abilities to complete tasks, promoting active engagement and rehabilitation. Conversely, lower parameter values provided greater assistance, adequate for more severe impairments. Thus, this framework allows the therapist to dynamically adjust the difficulty of the task in response to the patient’s changes in agency and capabilities (Figure 3C).

#### 3.1.3 Virtual environments and GUI

##### 3.1.3.1 Virtual environments

**FIGURE 4:**
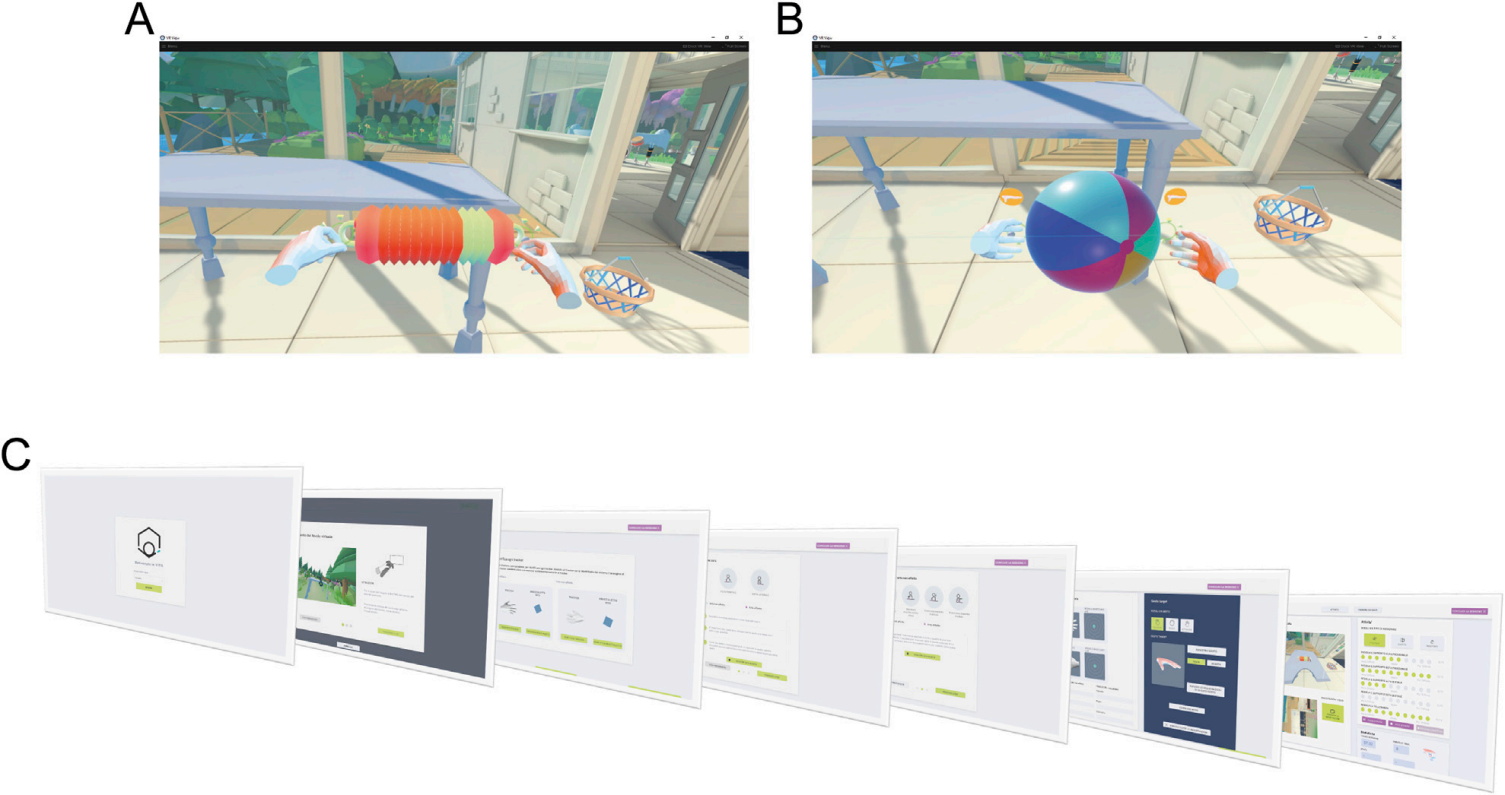
Virtual environment and GUI. The virtual environment consists of a room in a house with a desk and two different objects, a concertina **(A)** and a beach ball **(B)**. Participants are instructed to reach for these objects with both hands and grab them using the specified gesture—fingers closed for the concertina and fingers open for the beach ball. When the correct position and gesture are maintained for a set duration, the object progressively turns green. **(C)** Shows the sequential workflow of the therapist’s GUI.

The VVITA software facilitates bimanual upper-limb reaching tasks in an immersive VR environment. The virtual environment consists of three distinct scenarios: a house, a garden near the house, and a green space by a pond, designed to provide engaging and varied settings for rehabilitation based on real life interactions to reduce the cognitive load of learning new mechanisms. One scenario is randomly selected by the software each time it is launched. A table is positioned in front of the patient, and virtual representations of the patient’s hands, along with objects to be reached and grasped bimanually, are displayed. Two types of objects can be presented as targets in the virtual environment: a concertina (Figure 4A) and a ball (Figure 4B). All reaching and grasping tasks are designed to be symmetrical with respect to the target object, to ensure the effectiveness of the RLAL assistive guidance for the VMAL.

##### 3.1.3.2 GUI: setup of experimental parameters and rehabilitation protocol

To meet the therapists’ specifications and ensure a user experience aligned with their expectations, a dedicated GUI (Figure 4C) was developed to control experimental parameters and guide the execution of the rehabilitation protocol. First, the virtual environment is calibrated to the physical workspace by using HTC Vive controllers. During this step, the operator marks the corners of the real table to ensure precise alignment of the corresponding virtual table. Once this calibration is completed, the Myo armbands and the tracker, worn by the patient, are activated and recognized by the system, enabling it to distinguish between the less affected and more affected limbs. The patient then performs a series of calibrations tasks under the supervision of the operator, aimed at defining motor capabilities and tailoring the experimental parameters accordingly. First, a resting pose calibration is conducted to determine the baseline hand position. This is followed by a proximal calibration to establish maximum range of motion. Finally, during the distal calibration, EMG signals are recorded to train the gesture recognition model. After these preliminary steps, the operator selects the patient’s profile and customizes the rehabilitation approach by choosing one of three assistance modalities: stiff coupling, rubber band, or trajectories. Additionally, the software allows fine-tuning of four key parameters (α_p_, α_d_, β_p_, β_d_) to adjust task difficulty, provides performance metrics such as the number of successful trials, and securely stores patient-related data.

#### 3.1.4 Rehabilitation protocol and task design

Patients performed rehabilitation exercises with the VVITA system over the course of 1 month, with sessions scheduled three times per week, lasting 30 min each. During each session, the patient performs a series of movements grouped into blocks, with each block consisting of 12 bimanual reaching and grasping movements.

**FIGURE 5:**
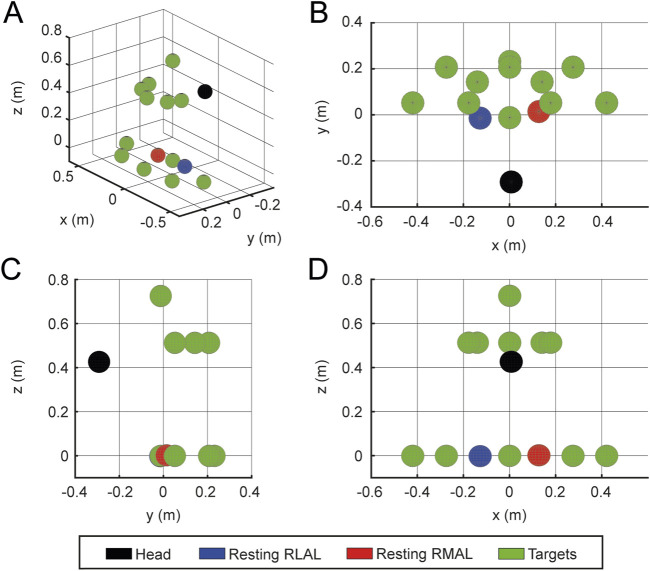
Target placement. Example target placements for $\beta_{p}=1$ . **(A)** 3D isometric view; **(B)** top view; **(C)** lateral view; **(D)** frontal view of the 12 targets (green spheres), the head (black sphere) and RLAL (blue sphere) and RMAL (red sphere) resting poses.

The target to be reached is randomly selected from the predefined set of locations (Figure 5). Target positions vary in height and lateral deviation relative to the initial recorded resting position of the hands on the table. Positions are defined as $x_{tar}=x_{tar}\left[r,\theta ,h,\beta_{p}\right]$ , where r is the radial distance from the starting hand position to the target center, θ is the target azimuth, and h is the target height. The distance parameter r is dynamically adjusted as a fraction of the maximum distance recorded during calibration ( $r=\beta_{p}\;r_{\max}$ ). The azimuth θ and height h of the targets are configured to span the entire calibration space, ensuring comprehensive coverage of the patient’s reachable workspace. Similarly, the muscle activation level required for successful hand gestures are determined by $\beta_{d}$ , with higher values requiring greater activation intensity to achieve a successful grasp.

Each trial begins with the patient’s hands resting comfortably on the table, close to the body, with elbows flexed at 90°. Midway through the session, the patients remove the head-mounted display and take a 3-min break. Target positions for each block are randomly selected (without replacement) from the 12 predefined positions. At the beginning of each session, the patients calibrate their range of movement by performing maximal motions to the boundaries of their workspace with both the less-affected and most-affected limbs. The maximum height and forward reach recorded during calibration are used to set the target placement distances for all subsequent trials.

The two target objects, the concertina and ball, are alternated throughout the session to require different hand gestures for each target. The concertina requires a fist gesture (fingers closed), while the ball requires a hand extension gesture (fingers opened). Both objects are equipped with two handles positioned on opposite sides, guiding the required hand placement for successful interaction. A reaching movement is considered successful when both virtual hands are within 0.05 m of the target handles.

Real-time feedback on hand placement and gesture success is provided within the VR environment. When the virtual hands are within the required tolerance, the handles’ color changes from orange to green. Additionally, a visual indicator of the required hand gesture disappears once the gesture is correctly performed. Upon meeting both position and gesture requirements, the object progressively turns green, visually signaling the required holding time (Figure 4A). Two types of auditory feedback are provided: a positive cue for successful completion of the task and a negative cue if the trial exceeds a 20-s time limit. The trial duration is limited to 20 s to prevent fatigue and to maintain task efficiency.

#### 3.1.5 Task adaptation and performance monitoring

At the end of each block, the therapist adjusts the proximal and distal agency ( $\alpha_{p}$ , $\alpha_{d}$ ) and capability ( $\beta_{p}$ , $\beta_{d}$ ) parameters based on the patient’s performance score $\pi_{j}=\pi \left(t_{1},\ldots ,t_{T_{j}}\right)$ , calculated for block j of $T_{j}$ trials. The performance score determines task difficulty adjustment:

• >90% Success Rate (≥11 successful trials): Task difficulty is increased.
• 70%–90% Success Rate (9–10 successful trials): Task difficulty remains unchanged.
• <70% Success Rate (≤8 successful trials): Task difficulty is decreased.

**FIGURE 6:**
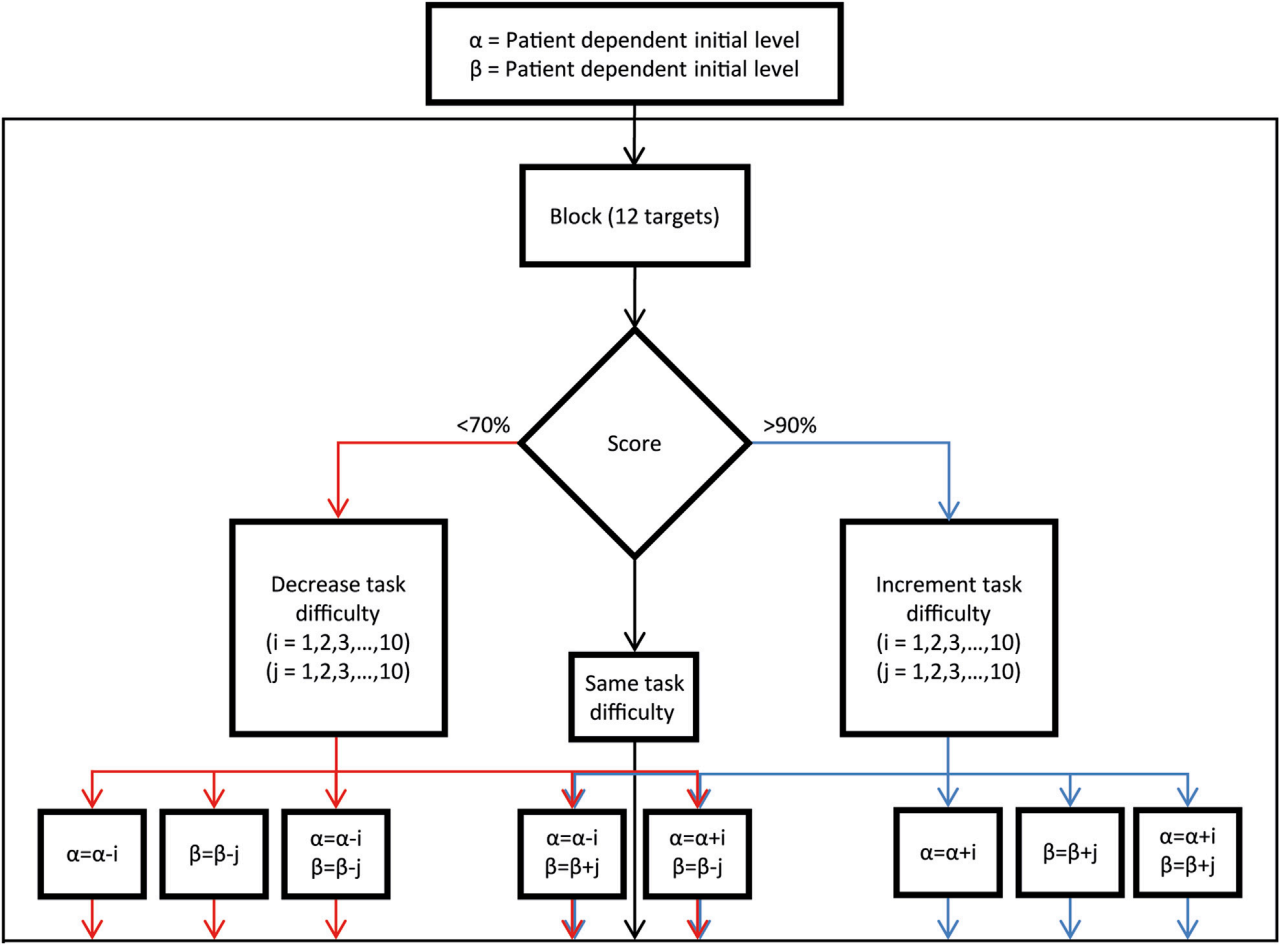
Assistance and difficulty parameters adjustment procedure. Decision block diagram illustrating the process for adjusting difficulty parameters based on the success performance of the previous reaching block (12 trials), as utilized by the operator.

This adaptive framework ensures that the task remains within an optimal challenge range, maintaining a success rate of 70%–90% to promote engagement and motor improvement throughout the rehabilitation process (Figure 6). The selection of the parameter to be adjusted, either to increase or decrease task difficulty based on the score, was made by the therapist, guided by clinical expertise and real-time observation of the patient’s needs.

#### 3.1.6 Challenges, limitations and solutions

During the development of the VVITA system, several challenges emerged, which were systematically addressed following the UCD approach. One key issue was the misalignment between the virtual and real tables, causing visuo-proprioceptive mismatches and unreachable targets. This was solved by enhancing the alignment procedure to ensure accurate calibration. Additionally, proximal assistance parameters sometimes led to unnatural movements in “stiff coupling” and “rubber band” modes; the “stiff coupling” method was refined as the default due to its closer resemblance to natural kinematics. Moreover, patients reported visual distractions from non-interactable objects and flickering in the virtual environment, prompting the removal of unnecessary objects and the introduction of a more neutral virtual hand color to improve embodiment. Calibration procedures also required adjustments, as fixed times led to inaccurate motion range estimates, and extended trial durations caused fatigue. These were resolved by introducing operator-controlled calibration and reducing trial durations from 30 to 20 s. Patients with limited functionality often compensated for impaired limb movements with excessive trunk motion, which therapists mitigated through physical interventions rather than more invasive solutions suggested like belts or additional tracking systems. Target placement issues were also addressed by introducing configurable target positions and sequences, along with a threshold to prevent unreachable target positions. These iterative improvements highlight the significance of integrating patient and therapist feedback in the system refinement, ultimately enhancing its usability, adaptability, and effectiveness in post-stroke rehabilitation.

### 3.2 Outcomes of the pilot study

In this section, clinical and instrumental results from four stroke patients recorded during the pilot study are reported. Outcomes from two chronic patients (E1 and E2) who performed 13 and 12 sessions respectively (including the first familiarization session) are presented in more detail. For the two subacute patients (E3 and E4) who performed 4 and 2 sessions respectively (including the first familiarization session), only feasibility and acceptability questionnaire responses are reported.

#### 3.2.1 Performance assessment

**FIGURE 7:**
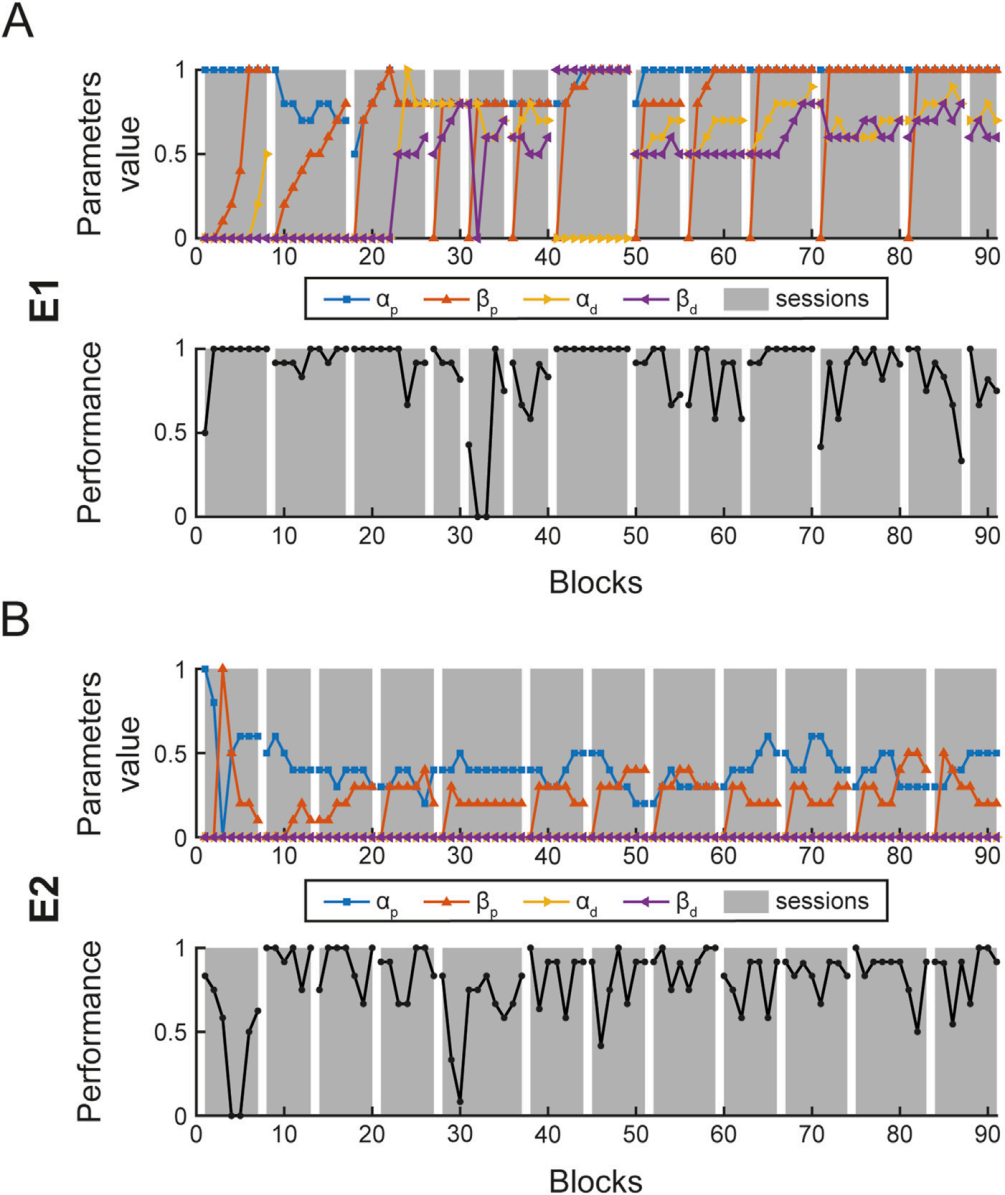
Block parameter selection and performance. Each panel shows the four assistance parameters ( $\alpha_{p},\beta_{p},\alpha_{d},\beta_{d}$ ) used for each block and the participants’ performance (number of successful trials over the number of trials) for patient E1 **(A)** and E2 **(B)**.

During each session, assistance parameters (α and β, proximal and distal) were adjusted at the end of each block according to the performance in that block, as described above (Figure 6). In Figure 7, the assistance parameters and the performance for each block across all the sessions are reported for patient E1 (A), who had a higher level of residual functionality and patient E2 (B), who had a lower level of residual functionality. As shown in the performance graphs, when performance was below 70% or above 90%, the difficulty was decreased or increased by adjusting the assistance parameters accordingly. Thus, the therapist was successful in keeping the level of performance around 80%. During the first session, a familiarization phase was performed in which the maximum level of α proximal and then β were set to let participants explore the software capabilities. A medium level of difficulty, based on participant performances and therapists’ observations, was then selected to start the therapy.

**FIGURE 8:**
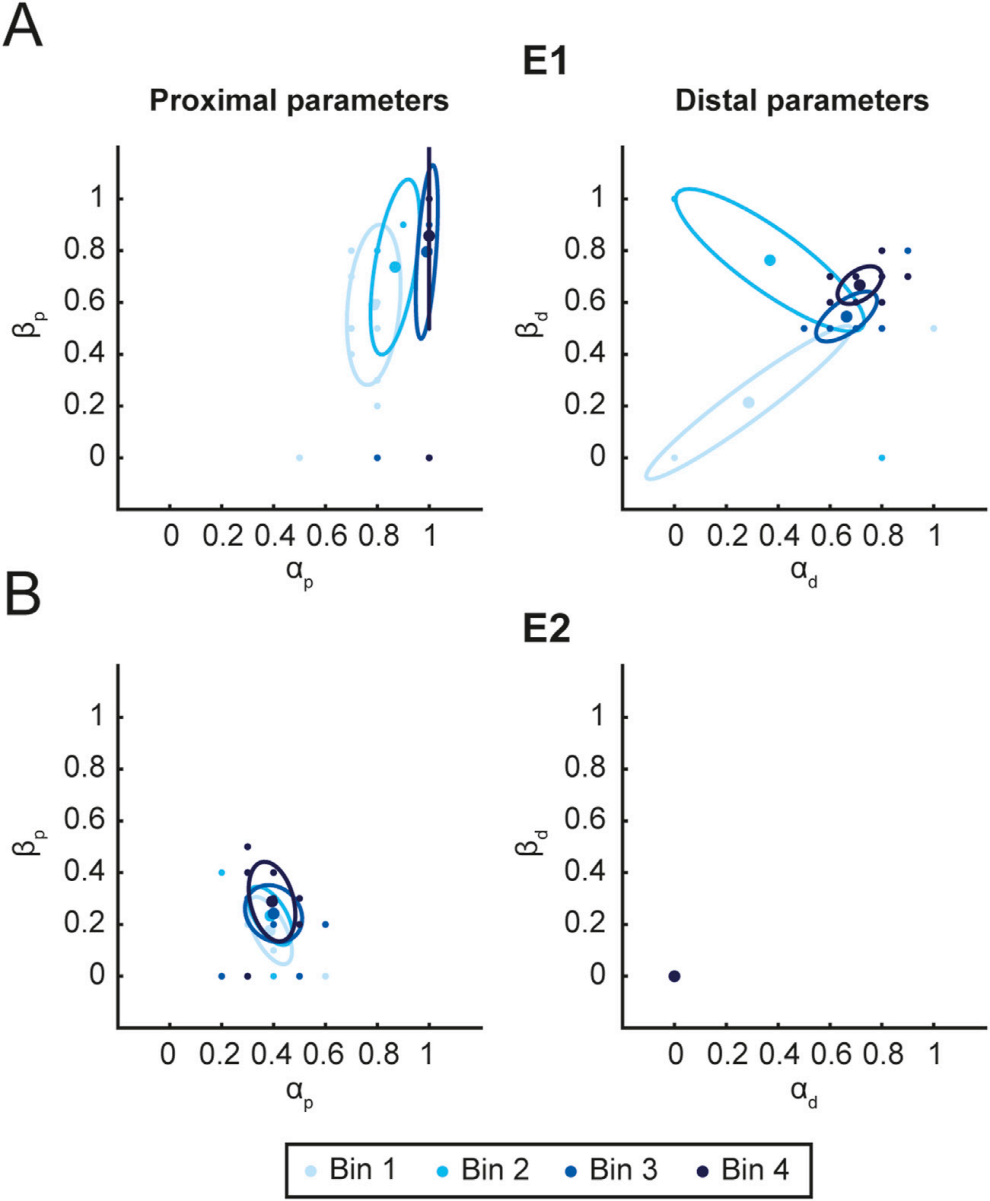
Parameters distribution. Figure shows the distribution of proximal (left column) and distal (right column) assistance parameters for E1 **(A)** and E2 **(B)**. For each time interval Bin (3 sessions), the mean value (ellipse center) and covariance (95% CI. ellipse) of α and β are reported. Color saturation represents the temporal evolution of the distribution.

Figure 8 shows the distributions of parameters used during the rehabilitation protocol for E1 (A) and E2 (B). For each patient, the distribution of the proximal (left column) and distal (right column) assistance parameters are shown. The figure highlights a clear difference between E1 and E2. For E1, the proximal parameters are both close to one (mean ± standard deviation (SD): $\alpha_{p}=0.91\pm 0.10$ ; $\beta_{p}=0.75\pm 0.11$ ), indicating less assistance. In contrast, for E2, who was more impaired than E1, both proximal parameters were lower than 0.6 (mean ± SD: $\alpha_{p}=0.39\pm 0.01$ , $\beta_{p}=0.23\pm 0.05$ ). Furthermore, for E1 the parameters overtime became closer to 1 than for E2.’ with ‘Furthermore, for E1 the parameters, over time, became closer to 1 than for E2. This indicates that, while maintaining the same performance level, E1 was able to perform the task with greater difficulty compared to E2. However, since the therapist could modulate task difficulty by adjusting both proximal and distal assistance parameters, we also computed an overall assistance index to summarize tasks difficulty.

**FIGURE 9:**
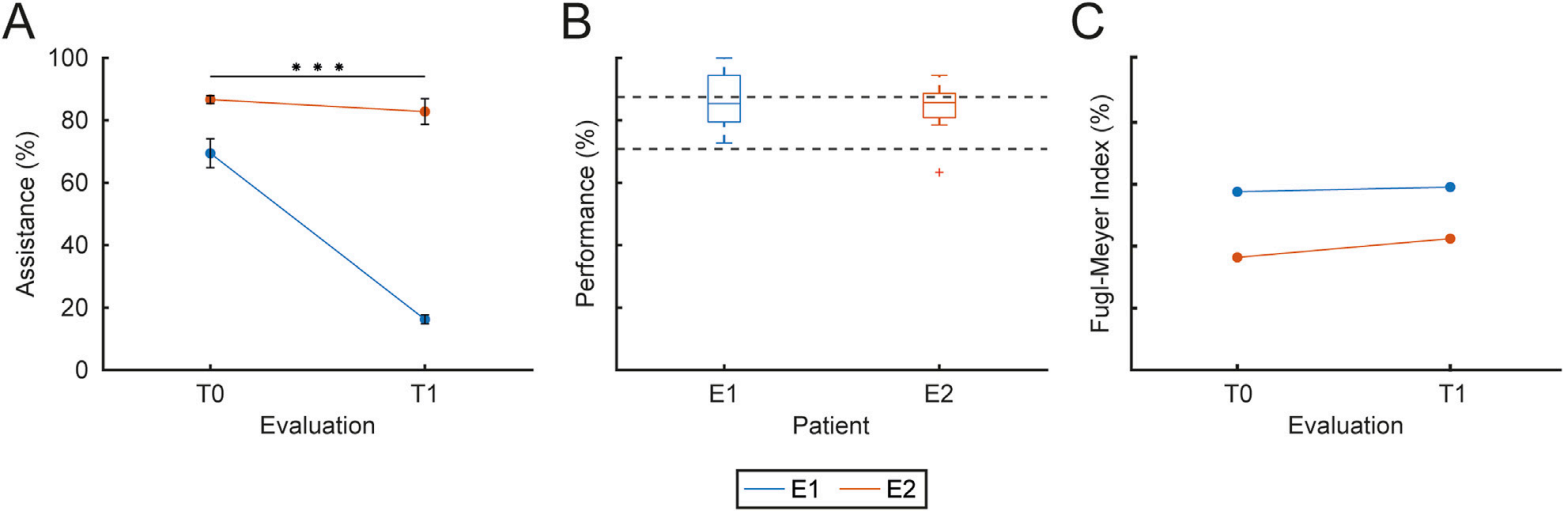
Training assistance, performance and clinical outcomes. The figure presents the assistance level, the overall performance and the clinical evaluation FM index for each patient (different colors). **(A)** Mean and SD of the assistance level provided to each patient at T0 and T1. **(B)** Overall performance, expressed as the percentage of successful trials over total trials for each patient. **(C)** Clinical evaluation of upper-limb motor function at T0 and T1, normalized as a percentage (with 66 corresponding to 100%), for each patient. Statistical significance between sessions is reported as *** for p<0.001 , ** for p<0.01 , and * for p<0.05 .

The overall assistance level $1-\frac{\alpha_{p}+\beta_{p}+\alpha_{d}+\beta_{d}}{4}$ decreased for both patients between T0 and T1 (Figure 9A, different colors). Assistance data of each patient for each block were fitted with the LMM model of Equation 1 ( $R^{2}=0.72$ ), which revealed a significant main effect of session ( p<0.001 ).

The therapist was instructed to change the assistance parameters in order to maintain performance at around 80% in each block. Figure 9B shows the overall performance for each patient. Performance was 87±9 (mean ± SD) for E1 and 84±8 for E2, confirming that the therapist correctly implemented the policy for maintaining a constant level of task difficulty.

Participants E1 and E2 were evaluated clinically at the beginning (*T*0) of the rehabilitation protocol and at the end (*T*1) using the Fugl-Meyer assessment scale, which has 3 points (index: 0, 1, 2) for each item of the upper-limb motor function assessment.’ with ‘Participants E1 and E2 underwent clinical evaluation at *T*0 and *T*1 using the Fugl-Meyer assessment scale, which employs a 3-point scale (0, 1, 2) for each item assessing upper-limb motor function. Figure 9C shows the evolution of the Fugl-Meyer motor function assessment index for each patient (different colors) for each evaluation ( T0 , T1 ), expressed as percentage of the maximum score (66 points). For E1 and E2 the motor function upper-limb score increased during the rehabilitation protocol from 38 to 39 (57.6%–59.1% of the maximum score), corresponding to an increase of 1.52% for E1 and from 24 to 28 (36.4%–42.4%), corresponding to an increase of 6.06% , for E2. However, when the Fugl-Meyer assessment index data were fitted with the LMM model of Eq. 1 ( $R_{2}=0.99$ ), the main effect of session was not significant ( p=0.14 ).

**FIGURE 10:**
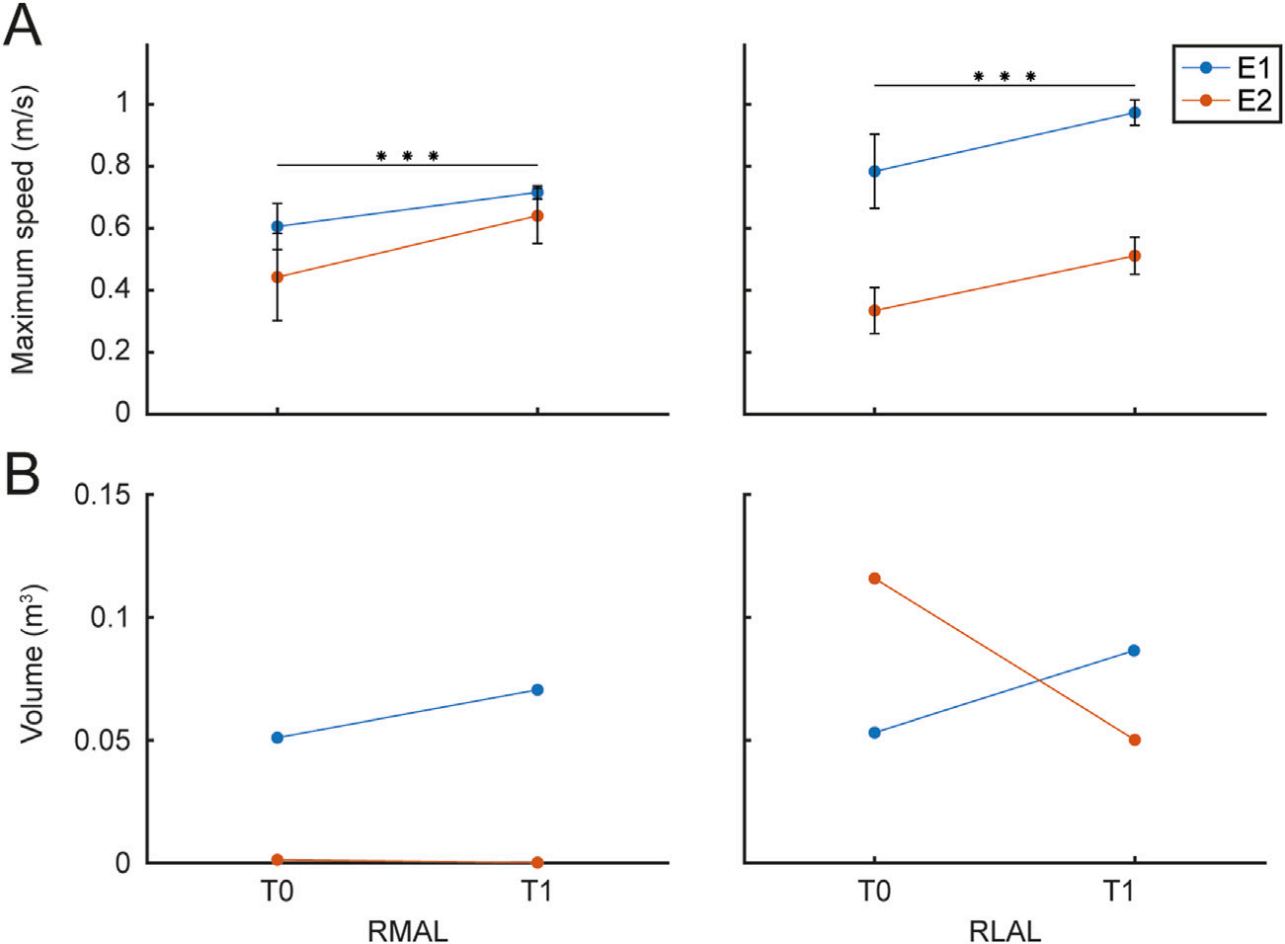
Maximum Speed and Movement Range. The figure presents the maximum speed and the volume of the maximum movement range achieved by each patient (different colors), evaluated during the rehabilitation protocol and the initial calibration procedure at T0 and T1 sessions. **(A)** The left panel shows the maximum speed for the RMAL, while the right panel shows the maximum speed for the RLAL. **(B)** The left panel displays the convex hull estimated for RMAL hand paths, while the right panel shows the convex hull for RLAL hand paths, both assessed during the initial calibration of maximum range movements.

Maximum speed was estimated for RMAL and RLAL in each trial to evaluate its evolution during the rehabilitation protocol. The average maximum speed values for each limb for all patients are shown in Figure 10A for T0 and T1. Maximum speed averaged within each session were fitted with the LMM model of Equation 1. (RMAL $R_{2}=0.92$ , RLAL $R_{2}=0.88$ ), which revealed a significant main effect of session for both limbs (RMAL p=0.047 , RLAL p<0.001 ).

Finally, to quantify the range of movement, an estimation of the volume of the convex hull of the trajectories recorded during the calibration procedure, performed at the beginning of each session, was obtained (Matlab, *convhull* function). Figure 10B shows the average RMAL (A) and RLAL (B) convex hull volume values for each hand path for the two patients for the first session T0 and the last session T1. Convex hull volume data were fitted with the LMM model in Equation 1 (RMAL $R_{2}=0.97$ , RLAL $R_{2}=0.09$ ), which revealed a non-significant main effect of sessions for both limbs (RMAL p=0.34 , RLAL p=0.59 ).

**FIGURE 11:**
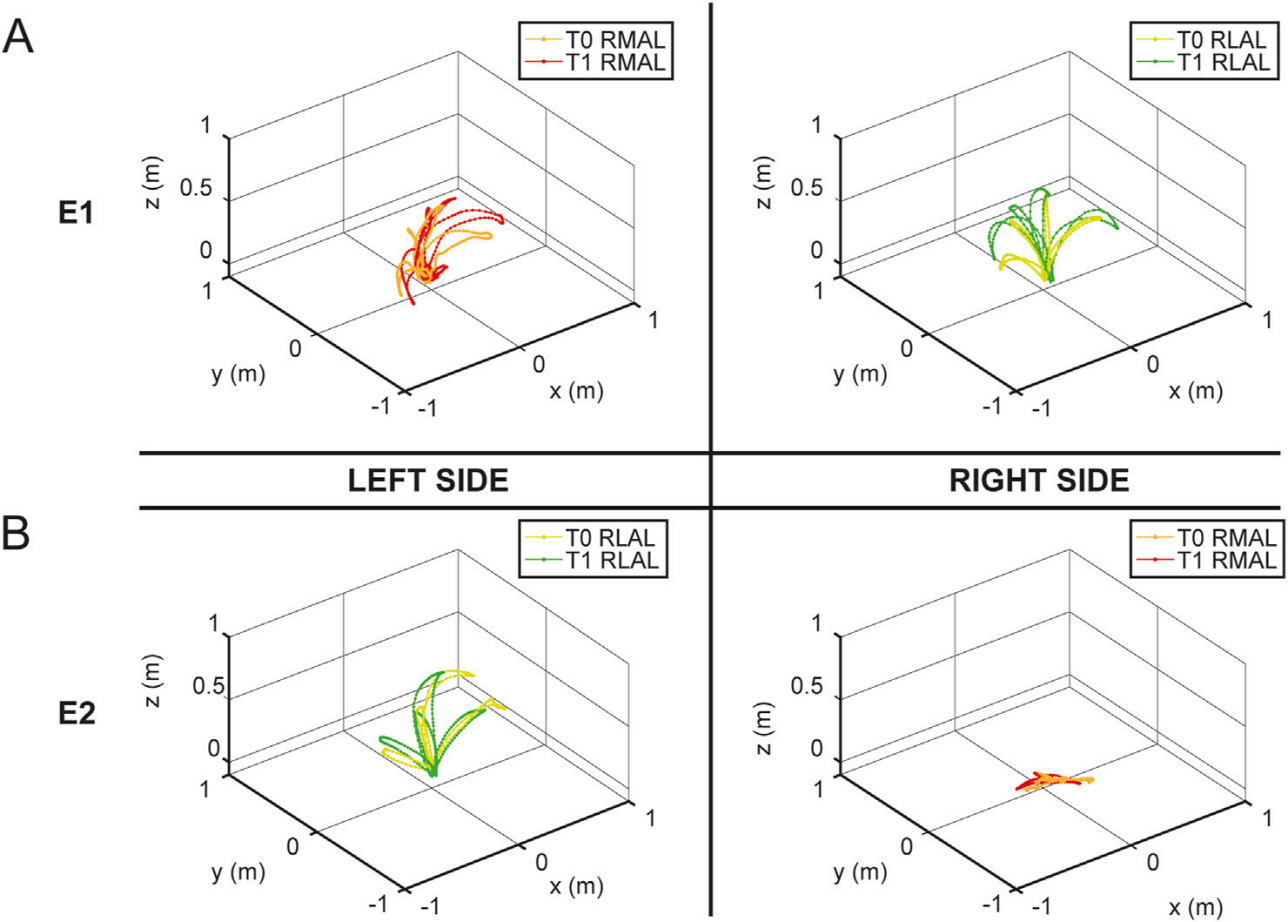
Hand trajectories during maximum range of motion calibration. The figure shows the spatial paths of the hand during the maximum range of motion calibration recorded at the beginning of the T0 and T1 sessions for E1 (panel **(A)**) and E2 (panel **(B)**), with both RMAL and RLAL. The left side of the figure represents left-hand movements, while the right side represents right-hand movements. RMAL trajectories are shown in red for T1 and in a lighter shade (orange) for T0. Similarly, RLAL trajectories are shown in green for T1 and in a lighter shade (yellow-green) for T0.

To highlight the differences in hand trajectories between the first and last sessions, Figure 11 illustrates the spatial paths recorded during the maximum range of motion calibration at the beginning of the T0 and T1 sessions for both E1 (panel A) and E2 (panel B). Left-hand movements are displayed on the left side of the figure, while right-hand movements are shown on the right. RMAL trajectories are represented in red for T1 and in a lighter shade (orange) for T0, whereas RLAL trajectories are shown in green for T1 and in a lighter shade (yellow-green) for T0.

#### 3.2.2 Usability assessment

**FIGURE 12:**
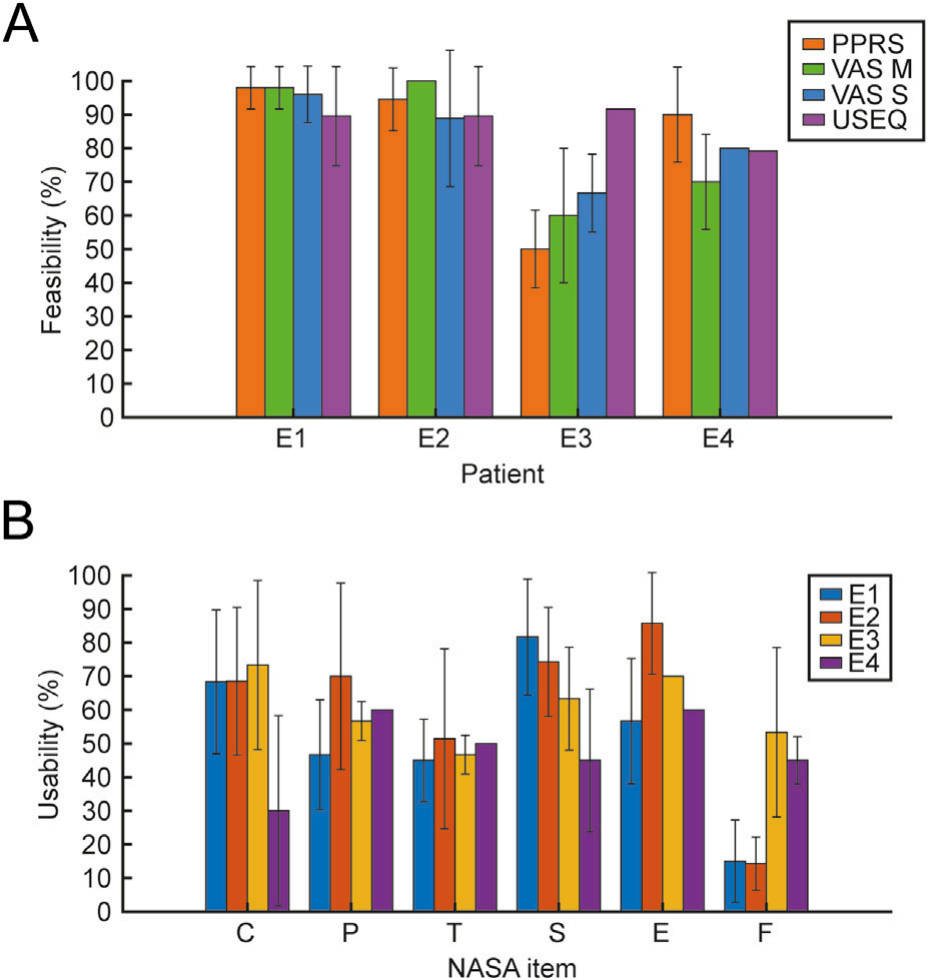
Feasibility and Usability scales. The figure shows the mean and SD of Feasibility **(A)** and Usability **(B)** values. For the feasibility outcome, the scales represented in different colors (PPRS, VAS 742 M, VAS S, and USEQ) are reported for the 4 patients. For usability, the 6 items of the NASA scale (C, cognitive demand; P, physical demand; T, temporal demand; S, perceived success; E, effort; F, frustration) are reported for the 4 patients, each represented in different colors.

All participants reported a good level of usability with the USEQ scale, even when motivation and perceived satisfaction were low as in the case of patient E3; further confirmed by the researcher’s evaluation through the administration of the PPRS scale (Figure 12A). Usability assessed by patients every session with NASA-TLX (Figure 12B), reported high levels of cognitive and physical demand; good levels related to the time domain (NASA_T) which indicates a general acceptability of the training, while, there are differences between the two chronic (E1 and E2) and subacute (E3 and E4) patients with respect to frustration (NASA_F) and self-assessment related to perceived success associated with the training (NASA_P).

## 4 Discussion

The VVITA system represents a significant advancement in stroke rehabilitation approaches based on virtual reality, myoelectric control, and exergames, developed using a rigorous UCD methodology. This approach actively involved patients, therapists, and clinicians, ensuring the system’s features directly address real-world rehabilitation challenges. The system’s integration of immersive VR, adaptive assistance algorithms, and EMG control sets it apart from traditional and earlier VR-based rehabilitation approaches. This discussion provides a critical analysis of the VVITA system’s outcomes, its development process, and its position in the landscape of stroke rehabilitation technologies.

Traditional rehabilitation approaches, such as mirror therapy and conventional VR systems, have demonstrated potential but are constrained by limited adaptability and personalization. Mirror therapy primarily relies on visual feedback to facilitate neural reorganization but cannot dynamically adjust to individual patient needs (Altschuler et al., 1999; Thieme et al., 2012). Similarly, conventional VR systems provide engaging environments but lack advanced control strategies to integrate patient-specific motor capabilities effectively (Bohil et al., 2011; Laver et al., 2017). VVITA addresses these limitations by combining immersive VR environments with dynamic task modulation based on real-time kinematic and EMG data, in which activity from the less and more affected arm are combined to control the virtual representation of the affected arm with adaptive degree of relative contribution. This approach overcomes the “learned non-use” phenomenon (Ballester et al., 2015) by enabling adaptive assistance tailored to the patient’s motor capabilities. Additionally, the use of EMG-driven interfaces promotes targeted motor recovery by integrating muscle activity into rehabilitation tasks (Song et al., 2013). These features position VVITA as a more engaging and effective alternative to traditional and earlier VR systems.

The VVITA system’s design and development were informed by 65 iterative interactions with clinical and technical stakeholders. These interactions highlighted key shortcomings in existing rehabilitation systems, such as insufficient adaptability and a lack of engaging, task-specific scenarios. Informed by these insights, VVITA incorporated gamified environments to sustain patient motivation (Burke et al., 2009). The UCD methodology ensured that every aspect of the system, from its adaptive algorithms to its user interface, was optimized for therapeutic effectiveness and patient usability (Meyer et al., 2019; Ríos-Hernández et al., 2021). Comparatively, VVITA’s UCD methodology builds on established practices in rehabilitation technology design. For instance, it has been demonstrated that the iterative, UCD approach highlights its value in developing effective lower-limb rehabilitation systems (Laffranchi et al., 2021; Semprini et al., 2022). Similarly, haptic robot-based telerehabilitation systems emphasize the importance of user-friendly interfaces and personalized therapy (Ivanova et al., 2017). VVITA extends these principles by integrating advanced EMG control and real-time task modulation, offering a novel approach to stroke rehabilitation.

Preliminary testing with four stroke patients, two chronic and two sub-acute, provided valuable insights into VVITA’s feasibility and effectiveness. Chronic patients exhibited higher engagement and notable improvements in motor performance, thanks to the benefits of tailored interventions (Torrisi et al., 2021; De Luca et al., 2024; De Pasquale et al., 2024). Instrumental assessments revealed significant improvements in maximum movement speed, correlating with clinical outcomes measured by the Fugl-Meyer Assessment. This correlation underscores the system’s potential to integrate objective metrics with subjective clinical evaluations, enhancing the assessment of rehabilitation effectiveness.

Interestingly, the pilot study revealed differences between patients in compliance and outcomes, with chronic patients responding more positively to the training protocol. This aligns with prior research which indicates that gamified VR interventions are particularly effective in maintaining motivation and improving motor function (Domínguez-Téllez et al., 2020). However, sub-acute patients faced challenges such as mood-related issues and medical complications, highlighting the need for further customization and support in this patient subgroup.

Both immersive and non-immersive virtual reality systems are now widespread in research and in clinical practice, however they do not have any type of control and adaptation to the paretic limb limiting the potentiality of the VR-assisted arm sensory motor rehabilitation. An important novelty of the present work is the myoelectric control of the paretic limb in virtual reality. This has two direct clinical positive consequences: i) it enhances the effectiveness of rehabilitation thanks to the greater integration of motor sensors consistent with the task; ii) it makes virtual reality exercise more adaptable to the patient’s motor function both intersession and with respect to the criteria for patient inclusion, allowing in fact to expand the beneficiaries. Moreover, therapists benefit from a real-time monitoring and feedback loop introduced by the system, which displays live kinematic, EMG, and performance metrics, enabling immediate adjustments to assistance levels and task parameters as therapy progresses.

The novelty of the VVITA approach lies in its integration of VR, EMG control, and adaptive assistance, features that distinguish it from existing rehabilitation systems. For example, traditional VR systems lacked the capability to dynamically adjust task difficulty based on real-time performance data (Ballester et al., 2015; Hoermann et al., 2017). Similarly, other systems focused on immersive environments but did not integrate advanced control strategies to modulate assistance levels or enhance task specificity (Lee et al., 2011; Laver et al., 2017; Ceradini et al., 2024; Mani Bharathi et al., 2024).

The VVITA system’s ability to combine kinematic and EMG data for personalized therapy represents significant improvement with respect to previous approaches presented in literature. Indeed, the importance of adaptive systems in achieving meaningful motor recovery aligns with findings in the literature (Sadarangani et al., 2017; Morone et al., 2019). However, further studies are needed to compare VVITA directly with other advanced systems, such as robotic exoskeletons or hybrid VR systems, to assess its clinical efficacy and cost-effectiveness.

Nonetheless, the very small sample size (four patients) is an important limitation of this study. Such sample size is appropriate for a pilot study assessing the feasibility of a novel approach but limits the generalizability of the findings. Therefore, the results should be interpreted with caution, and further research with larger, more diverse cohorts is necessary to validate these preliminary observations. Building on the promising results of the pilot study, a randomized controlled trial will evaluate VVITA’s clinical efficacy compared to conventional rehabilitation methods. Similarly to what is shown here, key outcomes will include clinical indices (e.g., Fugl-Meyer Assessment), kinematic performance metrics, and patient-reported satisfaction.

## 5 Conclusion

VVITA provides a novel and adaptable approach to stroke rehabilitation, addressing critical gaps in traditional and VR-based systems. By leveraging UCD principles, advanced control mechanisms, and engaging VR environments, the system holds significant potential to improve patient outcomes and set a new standard for adaptive rehabilitation technologies.

## Data Availability

The raw data supporting the conclusions of this article will be made available by the authors, without undue reservation.

## Abbreviations

VR, virtual reality; VITA, Virtual Therapy Arm; VVITA, Validation of the Virtual Therapy Arm; UCD, user-centered design; EMG, electromyographic; CT, conventional therapy; ADL, activities of daily living; MT, mirror therapy; GUI, graphical user interface; VMAL, virtual more affected limb; RMAL, real more affected limb; RLAL, real less affected limb; USEQ, User Satisfaction Evaluation Questionnaire; VAS, Visual Analogical Scale; PPRS, Pittsburgh Participation to rehabilitation Scale; NASA-TLX, NASA Task Load Index; NASA-TLX, National Aeronautics and Space Administration Task Load Index; FMA, Fugl-Meyer Assessment; T0, beginning of the rehabilitation protocol (baseline assessment); T1, end of the rehabilitation protocol (post-treatment assessment); LMM, linear mixed model; α, agency parameter; β, capability parameter.
